# Diatomite/Cu/Al layered double hydroxide hybrid composites for polyethylene degradation

**DOI:** 10.1039/c9ra10940d

**Published:** 2020-03-06

**Authors:** Nengshuo Fu, Shuhua Zhang, Yingying Ma, Zhuo Yang, Weijun Liu

**Affiliations:** College of Chemistry and Chemical Engineering, Shanghai University of Engineering Science Shanghai 201620 China zsh_7474@126.com; College of Mechanical and Automotive Engineering, Shanghai University of Engineering Science Shanghai 201620 China

## Abstract

A diatomite/Cu/Al layered double hydroxide hybrid composite (DI-LDH) was synthesized using the hydrothermal method. The synthesized DI-LDH composites were characterized *via* X-ray diffraction (XRD), Fourier transform infrared spectroscopy (FT-IR), scanning electron microscopy (SEM) and the Brunauer–Emmett–Teller (BET) method. Polyethylene degradation over DI-LDH was studied in a batch reactor. DI-LDH showed layered structures, indicating that the diatomite/Cu/Al double hydroxide hybrid was successfully synthesized. A significant decrease in the degradation temperature and the released amounts of CO and CO_2_ was observed in the DI-LDH catalytic degradation reaction, which indicated that DI-LDH was helpful for the polyethylene degradation reaction. The X-ray photoelectron spectroscopy (XPS) results suggested that the reaction of Cu^2+^ → Cu^+^ occurred in polyethylene catalytic pyrolysis, which resulted in the decrease in the released CO amount. DI-LDH may be a potential environmental catalyst that can be applied to treat LDPE waste.

## Abstract 2

DI-LDH that can reduce the pyrolysis temperature of LDPE and the release of carbon monoxide (CO) and carbon dioxide (CO_2_).

## Graphical abstract

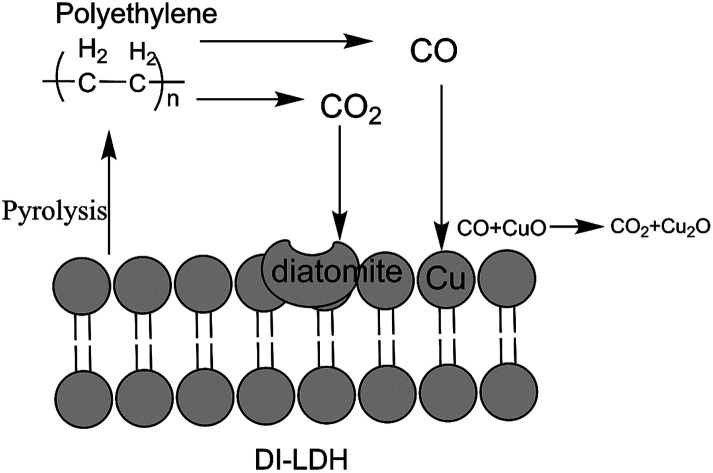

## Introduction

Plastic materials have been widely used in the world due to their low price, strong production capacity and simple processing technology. The consumption of these materials has been increasing, which may also cause serious environmental problems.^1^ Landfill, incineration, and mechanical and chemical recovery are the treatment methods for plastic waste. In the landfill disposal method, plastic waste is buried.^2^ It is usually not biodegradable, and landfill plastic waste will degrade after hundreds of years. Therefore, a lot of space is needed, and the available free space is exhausted every day. In the incineration method, plastic waste is burned. Depending on the nature of the plastic waste, highly toxic chemicals are produced in the exhaust gases. Therefore, it is harmful to the human health and requires an additional exhaust gas purification treatment device.^3^ A recycling method reduces energy use and raw material consumption. Mechanical recycling of plastic waste is the simplest and relatively inexpensive treatment compared to other methods. However, there is a certain degree of loss in material quality and performance. Pyrolysis of polymers seems to be the most promising in terms of environmental safety and valuable chemical recovery.^4^

The pyrolysis of plastic materials can be divided into catalytic pyrolysis and non-catalytic pyrolysis. Catalytic pyrolysis is clearly advantageous over non-catalytic pyrolysis.^5^ In the presence of a catalyst, pyrolysis starts at a lower temperature and the residence time of the reaction is very short.^6^ Lower temperatures and residence times reduce the energy consumption of the pyrolysis process. The degradation products are mainly coke and gaseous and liquid products; these are potential fuels and raw materials for the petrochemical industry. In addition, in non-catalytic pyrolysis, the product has a broad molecular weight distribution and requires further processing to obtain higher quality carbon-containing compounds.^7^

Aluminosilicates and mesostructured materials are the common catalysts used in polymeric material cracking reactions. Diatomite and hydroxides are all mesostructured materials.^8^ Diatomite is a fossil of single-cell aquatic plants (algae), which consists of a porous diatom skeleton and an Si–O tetrahedral interconnected network with excellent features such as low density, high surface area and large adsorption capacity.^9–13^ Layered double hydroxides (LDH) which are one type of layered materials with many outstanding catalytic behaviors, such as activity, selectivity and stability of active sites that have been extensively studied.^14–21^ LDH has been applied as an adsorbent and precursor of well-mixed oxides in various catalytic reactions.^20^ CuMgAl-LDH@SiO_2_ nanosheets were prepared by Guoqing Cui and co-workers, which enhanced the catalytic activity in phenol hydroxylation.^22^ Guoqing Zhao and co-workers prepared diatomite/ZnFe layered double hydroxide hybrid composites by a coprecipitation method, which enhanced the photocatalytic degradation of organic pollutants.^8^ Hydroxides and diatomite hybrid composites can have better catalytic activity compared to diatomite or Cu/Al-LDH.

In this study, the primary goal and tasks were to prepare catalysts that can reduce the pyrolysis temperature of LDPE and the release of carbon monoxide (CO) and carbon dioxide (CO_2_). The synthesis of the diatomite/Cu/Al layered double hydroxide hybrid composite (DI-LDH) was studied, and the catalytic performance of diatomite, Cu/Al layered double hydroxide (Cu/Al-LDH) and DI-LDH as well as the release of CO and CO_2_ in the LDPE pyrolysis reaction was investigated. The DI-LDH hybrid composites with good structures and catalytic properties were synthesized. Diatomite was successfully inserted into LDH. In the process of LDPE thermal cracking, DI-LDH had strong catalytic activity to reduce the generation of CO, and the use of diatomite was conducive to the combustion of LDPE and the release of CO_2_. As presented in this paper, the prepared DI-LDH catalyst has a good application value and economic value in the pyrolysis of plastic materials, which can significantly alleviate the problem of white pollution caused by the use of plastic products.

## Experimental

### Materials

Absolute ethanol, Cu(NO_3_)_2_·3H_2_O, Al(NO_3_)_3_·9H_2_O, di-*n*-octyl phthalate and urea were of reagent grade. Low-density polyethylene (LDPE) was used in the pellet form, and diatomaceous earth was chemically pure.

### Preparation of the diatomite/Cu/Al layered double hydroxide hybrid composite (DI-LDH)

The hydrothermal method was used to prepare the DI-LDH sample. In a typical synthesis method, a solution A containing Cu(NO_3_)_2_·3H_2_O (9.00 mmol), Al(NO_3_)_3_·9H_2_O (4.50 mmol), urea (12.00 mmol) and deionized water (45 mL) was prepared. Then, this solution was dropwise added with stirring to a solution B containing diatomaceous earth (3 mmol) and absolute ethanol (10 mL). The resulting homogeneous solution was then transferred to a 100 mL polytetrafluoroethylene (PTFE)-lined stainless-steel autoclave and the hydrothermal synthesis was conducted at 110 °C for 18 h. The final mixture was then filtered and washed with deionized water to recover the product as a powder. After washing, the product was dried at 100 °C for 12 h.

### Catalytic LDPE pyrolysis

Pyrolysis reactions of LDPE were conducted in a definite air tube furnace. The method used for preparing different catalysts based on LDPE is shown in Table 1. In a typical experiment, LDPE was mixed with the catalyst using a plastic mixer and then was compression-moulded into ∼2 mm-thick sheets using an electrically heated hydraulic press in a standard mould at 150 °C; di-*n*-octyl phthalate was used as the plasticizer (15 g). The LDPE cracking system comprised a flue gas analyser, a temperature-controlled tube furnace and a nitrogen bottle. Then, the sample (2 g) was placed in the reactor and heated to 450 °C or 500 °C at a heating rate of 20 °C min^−1^ in air for 2 h. After the completion of the reaction, a nitrogen flow was used to sweep the volatile products from the reactor. The gas leaving the reaction system was then collected in a gas–liquid separator and analysed using a flue gas analyser. The conversion is equal to 100% minus the difference between the pyrolysis residual mass of LDPE and the mass of LDPE before reaction:Conversion (%) = 100% − mass of coke collected after burning/mass of LDPE fed into the reactor × 100%

**Table 1:** Method used for preparing LDPE-based DI-LDH, Cu/Al-LDH and diatomite

Samples	LDPE (g)	DI-LDH (g)	Cu/Al-LDH (g)	Diatomite (g)
LDPE	100	—	—	—
LDPE/DI	100	—	—	2
LDPE/LDH	100	—	2	—
LDPE/DI-LDH	100	2	—	—

### Characterization

X-ray diffraction (XRD) analysis was performed on a D2 Phaser (Bruker, Germany) instrument at 35 kV and 30 mA using CuKα radiation in the 2*θ* range of 5–80° to determine the structure of DI-LDH. The surface functional groups of DI-LDH were investigated using Fourier transform infrared spectroscopy (FTIR) (AVATAR380, Nicolet, USA) in the wave number range of 400–4000 cm^−1^. The morphology and microstructures of DI-LDH were studied by scanning electron microscopy (SEM, JEOL-4800). N_2_ adsorption–desorption isotherms were obtained using a Micromeritics ASAP 2460 instrument to study the surface area, pore volume (VP) and pore diameter (DP) of the samples. Thermogravimetric (TGA) analysis was conducted at a heating rate of 20 °C min^−1^ in the temperature range of 50–600 °C in air. The flue gas analyser used was an MRU air analyser MGA 5-061161, Germany. X-ray photoelectron spectroscopy (XPS) experiments were performed on a Thermo ESCALAB 250XI device.

## Results and discussion

**Fig. 1:**
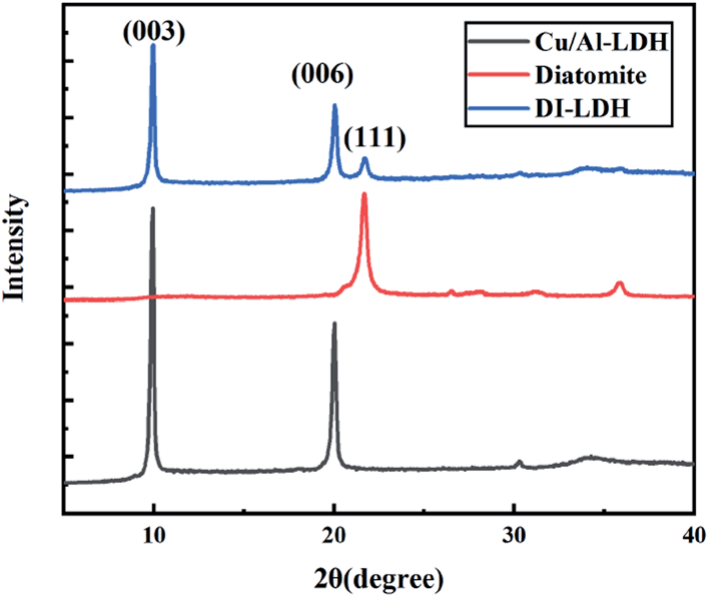
XRD patterns of Cu/Al-LDH, diatomite and DI-LDH.

Fig. 1 shows the XRD patterns of DI-LDH, diatomite and Cu/Al-LDH. Sharp and symmetrical reflections at 9.96°, 20.05° and 21.71° corresponding to the (111) diffraction of diatomite and the (003) and (006) crystal planes of Cu/Al-LDH can be observed in the XRD pattern of DI-LDH.^19^ The amorphous phase of opal (SiO_2_·*n*H_2_O) is reflected by the peak at 21°, which can be seen in the XRD pattern of diatomite.^9^ The typical characteristic peaks of hydroxides at 9.96°, 20.05° and 30.24° are attributable to the Cu/Al-LDH structure, which is similar to the result we previously reported.^16^ Thus, we conclude that diatomaceous earth has no impact on the hydrothermal preparation of copper aluminium hydroxides. The crystal form of diatomaceous earth did not vary under the hydrothermal experimental conditions.

**Fig. 2:**
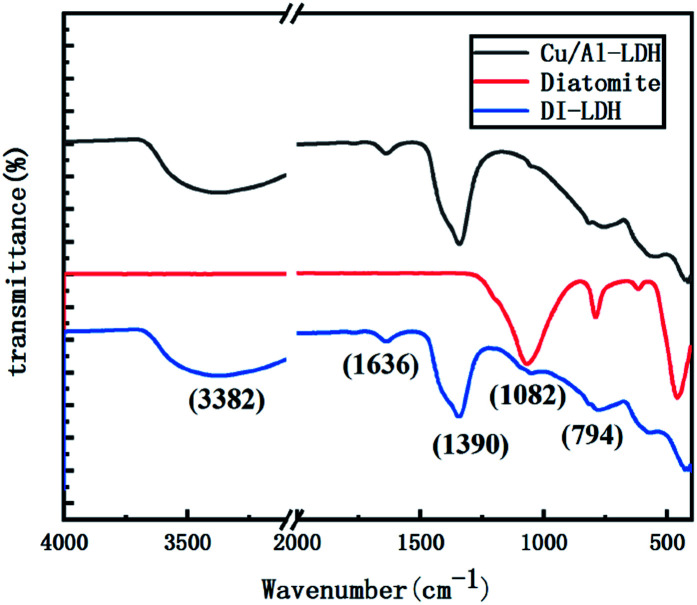
FTIR spectra of diatomite, Cu/Al-LDH and DI-LDH.

Fig. 2 shows the FTIR spectra of DI-LDH, diatomite and Cu/Al-LDH. The broad and strong absorption peak at ∼3382 cm^−1^ along with the bending mode at 1636 cm^−1^ can be attributed to the OH^−^ stretching vibration in the layers of Cu/Al-LDH and interlayer water molecules.^23^ The band at ∼1390 cm^−1^ is attributed to the NO_3_^−^ anions in the interlayer, indicating the presence of NO_3_^−^ in the inside of DI-LDH. The bands at 1082 cm^−1^ and 794 cm^−1^ can be assigned to the stretching vibration of Si–O–Si and the stretching vibration of Si–OH, respectively. These results indicated that diatomite was connected with Cu/Al-LDH *via* the hydroxyl silicon groups.

**Fig. 3:**
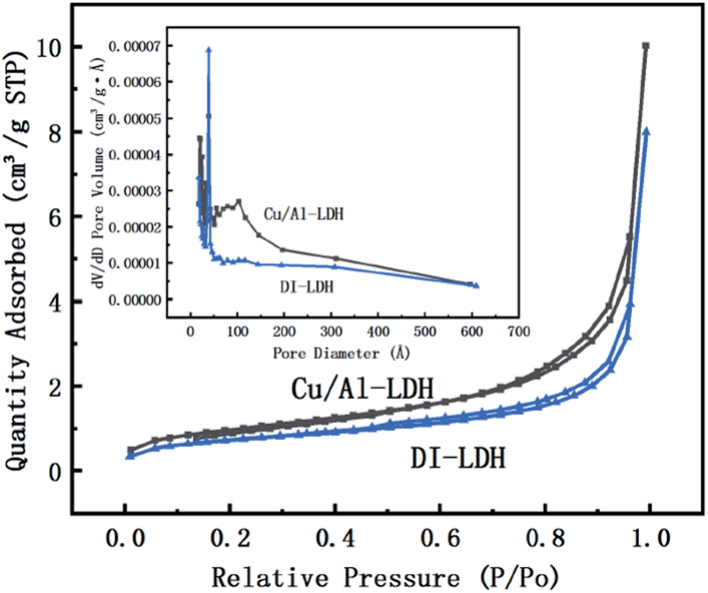
N_2_ adsorption–desorption isotherms and pore size distribution curves.

The specific surface area and pore structure of the catalysts were investigated by N_2_ adsorption–desorption isotherms, and the results are shown in Table 2 and Fig. 3. From Table 2, it can be determined that the pore size of diatomite is considerably lower than that of LDH, and the pore volume and surface area of DI-LDH decrease compared to those of Cu/Al-LDH, which indicates that diatomite has been inserted into LDH. DI-LDH exhibited type II isotherms, as shown in Fig. 3. These results indicated that DI-LDH comprised mesoporous structures and diatomaceous earth was inserted into LDH.

**Table 2:** Physical properties of the catalysts

Catalyst	Pore diameter (Å)	Pore volume (cm^3^ g^−1^)	Surface area (m^2^ g^−1^)
LDH	174	0.016	3
Diatomite	83	0.002	1
DI-LDH	203	0.012	2

**Fig. 4:**
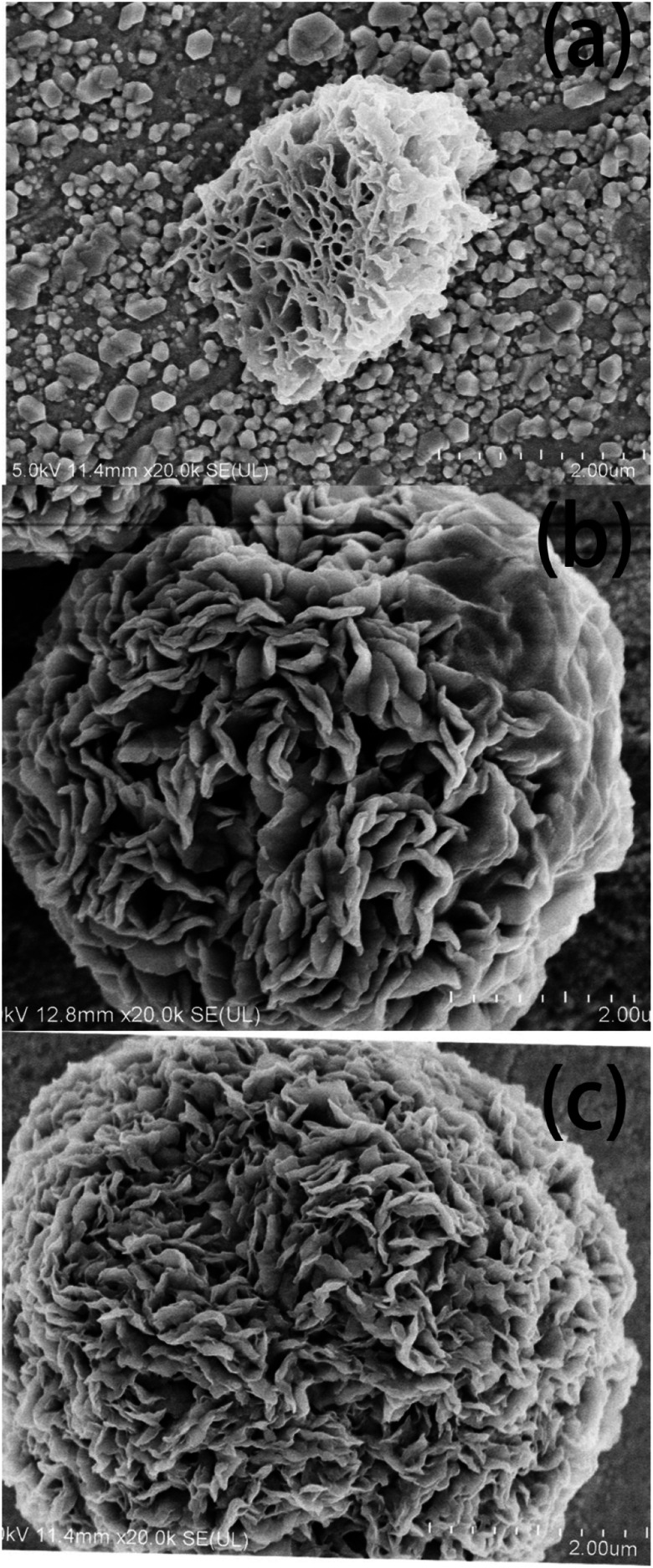
SEM images of (a) diatomite, 2 μm; (b) Cu/Al-LDH, 2 μm; and (c) DI-LDH, 2 μm.

Fig. 4 shows the SEM images of the diatomite-2 μm, Cu/Al-LDH-2 μm and DI-LDH-2 μm samples, which show the porous structure of diatomite and the flower structure of Cu/Al-LDH-2 μm; this is attributed to the presence of multiple irregular and randomly arranged sheets, exhibiting layered structures within the crystal. The SEM image of the DI-LDH-2 μm sample shows layered structures with a layer spacing smaller than that of Cu/Al-LDH, which indicates that diatomite has been inserted into Cu/Al-LDH. These results showed that diatomaceous earth was inserted into LDH.

**Fig. 5:**
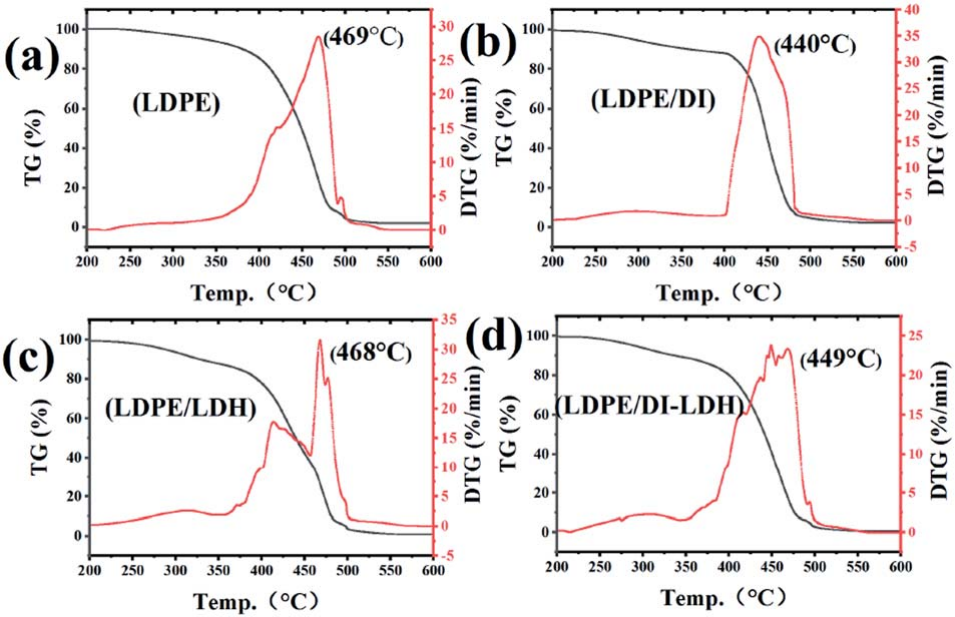
TG–DTG curves of (a) LDPE, (b) LDPE/DI, (c) LDPE/LDH and (d) LDPE/DI-LDH.

The quality of coke and conversion and the TG–DTG curves are provided in Table 3 and Fig. 5, respectively. According to the results summarised in Table 3, the conversions for LDPE/DI-LDH and LDPE/DI are higher than those for LDPE and LDPE/LDH at 450 °C, which suggests that diatomite and DI-LDH contribute to the LDPE pyrolysis. Two distinct weight loss stages can be observed in the TG–DTG curves of LDPE, LDPE/LDH and LDPE/DI-LDH. The first region ranges from 250 to 400 °C, and the second one is in between 400 and 500 °C. The first stage can be attributed to the volatilization of the plasticiser and Cu/Al-LDH, and the second is attributed to the degradation of LDPE molecular chains. The maximum degradation temperatures for LDPE, LDPE/DI, LDPE/LDH and LDPE/DI-LDH were determined to be 469, 440, 468 and 449 °C, respectively. These results are consistent with those included in Table 3 regarding the quality of coke and conversion, which indicates that DI-LDH is suitable for LDPE pyrolysis.

**Table 3:** Quality of coke and conversion^a^

Sample	Temperature/°C	Coke/g	Conversion/%
LDPE	450	0.78	61
LDPE	500	0.01	99
LDPE/DI	450	0.05	97
LDPE/DI	500	0.05	97
LDPE/LDH	450	1.10	45
LDPE/LDH	500	0.03	98
LDPE/DI-LDH	450	0.05	97
LDPE/DI-LDH	500	0.03	98

a All experimental samples (2 g) were heated in a definite air tube furnace for 2 h at a heating rate of 20 °C min^−1^.

**Fig. 6:**
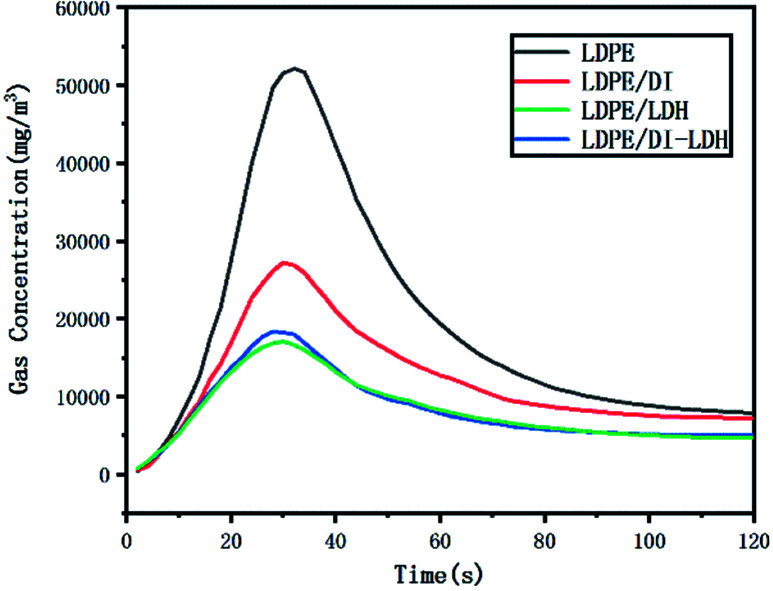
CO concentration curves for the pyrolysis of LDPE, LDPE/DI-LDH and LDPE/LDH at 450 °C.

**Fig. 7:**
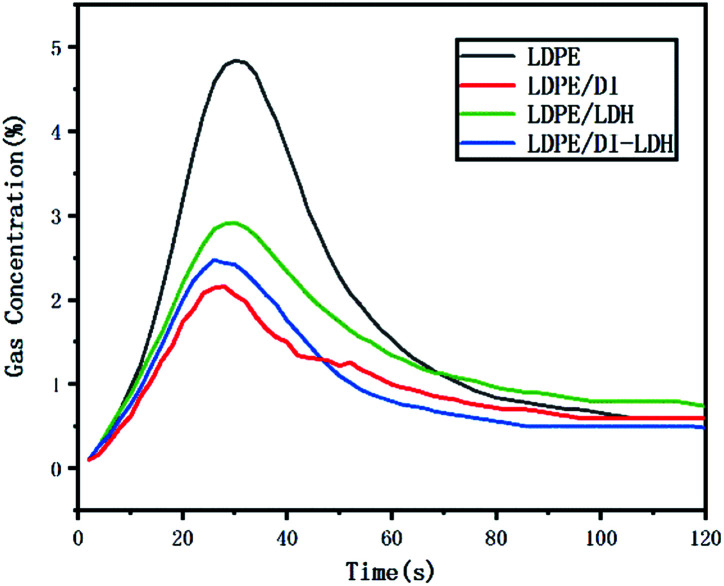
CO_2_ concentration curves for the pyrolysis of LDPE, LDPE/DI-LDH and LDPE/LDH at 450 °C.

The released amounts of CO and CO_2_ for LDPE, LDPE/LDH and LDPE/DI-LDH are shown in Fig. 6 and 7, respectively. CO and CO_2_ were produced in the reaction of LDPE degradation. The released amounts of CO for LDPE/LDH and LDPE/DI-LDH underwent a sharp decline compared to that of LDPE, which indicated that Cu/Al-LDH and DI-LDH were suitable for decreasing the release of CO. The released amount of CO_2_ for LDPE/DI-LDH was higher than that for LDPE/DI, which could be attributed to the reaction of CO and DI-LDH.

**Fig. 8:**
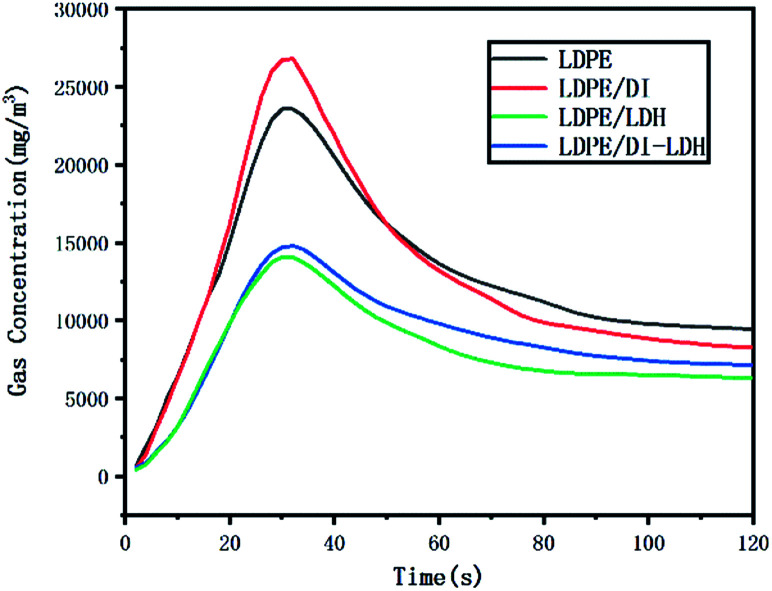
CO concentration curves for the pyrolysis of LDPE, LDPE/DI-LDH, LDPE/LDH and LDPE/DI at 500 °C.

**Fig. 9:**
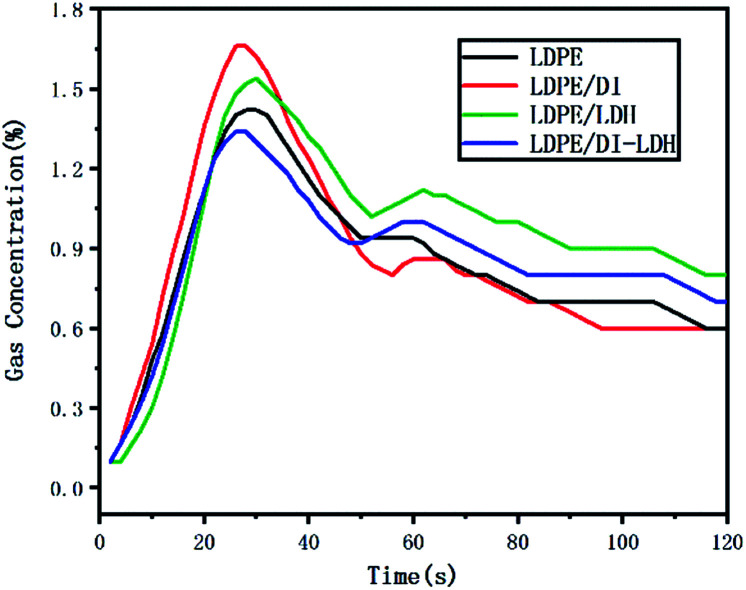
CO_2_ concentration curves for the pyrolysis of LDPE, LDPE/DI-LDH, LDPE/LDH and LDPE/DI at 500 °C.

The LDPE, LDPE/DI, LDPE/DI-LDH and LDPE/LDH samples were completely burned at 500 °C, as can be extracted from Table 3. As shown in Fig. 8, the maximum CO concentration for LDPE/DI-LDH decreases by 35% compared to that for LDPE, indicating that DI-LDH contributes to a decrease in the CO release. Moreover, the released amounts of CO and CO_2_ for LDPE/DI were higher than those for LDPE, which suggested the suitability of diatomite for LDPE pyrolysis by producing higher CO and CO_2_ emissions. The released amount of CO_2_ for LDPE/LDH was higher than that for LDPE due to the reaction of CO with Cu/Al-LDH to form CO_2_. Moreover, the released amount of CO_2_ for LDPE/DI-LDH was the lowest, indicating that DI-LDH decreased the CO_2_ release. From Table 3, it can be found that the conversion of LDPE/LDH at 450 °C is only 45%, whereas that of LDPE/DI-LDH is 97%; this difference indicates that the best catalyst at 450 °C is DI-LDH. From Fig. 9, it is clear that at 500 °C, the CO_2_ emissions for LDPE/DI-LDH are lower than those for LDPE/LDH, which confirms DI-LDH as the best catalyst. To summarize, these results show that DI-LDH is suitable for decreasing the release of CO and CO_2_.

**Fig. 10:**
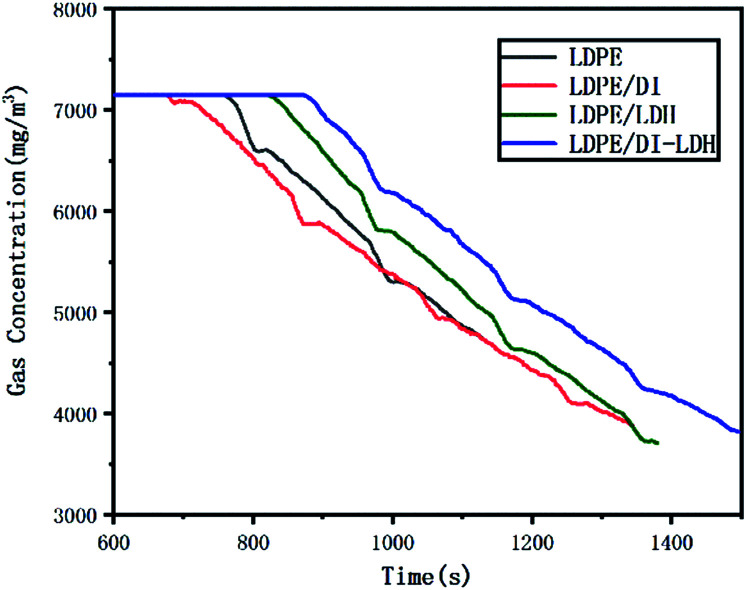
CH_4_ concentration curves for the pyrolysis of LDPE, LDPE/DI-LDH, LDPE/LDH and LDPE/DI at 500 °C.

Fig. 10 shows the CH_4_ concentration curves for LDPE, LDPE/DI, LDPE/LDH and LDPE/DI-LDH. The CH_4_ emissions for LDPE/DI-LDH were higher than those for the other catalysts because of the lower CO and CO_2_ emissions for LDPE/DI-LDH. The CH_4_ emissions for LDPE/DI were the lowest, which could be attributed to the higher CO and CO_2_ emissions for LDPE/DI. These results indicated that DI-LDH produced more CH_4_ during LDPE pyrolysis at 500 °C.

**Fig. 11:**
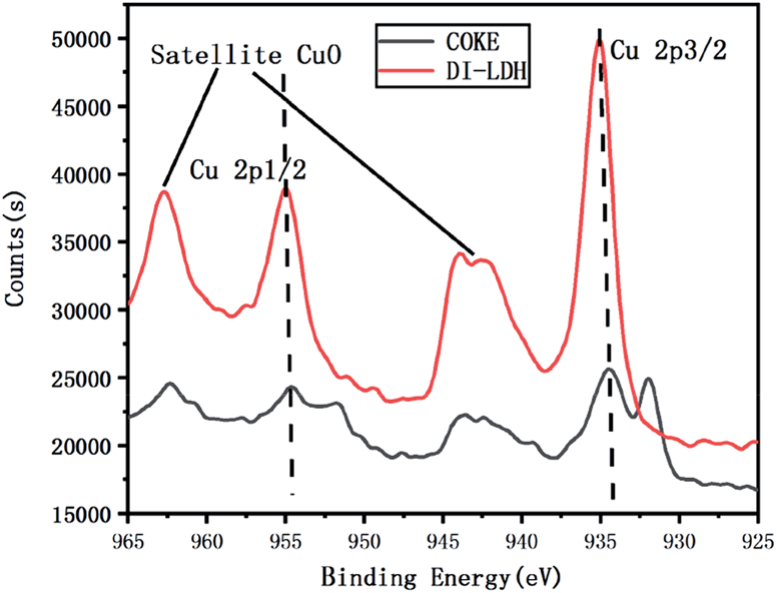
XPS spectra of Cu 2p of DI-LDH and coke from the pyrolysis of LDPE/DI-LDH at 450 °C.

The coke obtained from the pyrolysis of LDPE/DI-LDH at 450 °C and DI-LDH were subjected to XPS analysis to elucidate their chemical state and composition. Fig. 11 shows the XPS spectra in the Cu 2p region of the DI-LDH sample and coke obtained from the pyrolysis of LDPE/DI-LDH at 450 °C; the peak at the binding energy of 935 eV corresponds to Cu 2p_3/2_, while the peak at the binding energy of 954 eV corresponds to Cu 2p_1/2_.^24^ The shake-up peak at the binding energy of 943 eV suggests the existence of Cu^2+^ ions.^25^ The Cu 2p_3/2_ peak of DI-LDH shifts toward a higher binding energy, and its shake-up peak is more obvious compared to that of coke obtained from the LDPE/DI-LDH pyrolysis at 450 °C, indicating the presence of more Cu^2+^ ions on the surface of the DI-LDH catalyst. The satellite peaks at the binding energies of 943 and 962 eV indicate the presence of copper as Cu(ii) (*i.e.*, CuO phase) in DI-LDH and coke obtained from the LDPE/DI-LDH pyrolysis at 450 °C. The Cu-2p_3/2_ peak at 932 eV suggests that copper exists as Cu(i) (*i.e.*, Cu_2_O phase).^26^ This result indicates that the reaction CuO + CO → CO_2_ + Cu_2_O may occur on the surface of the DI-LDH catalyst.

## Conclusions

DI-LDH hybrid composites having excellent structural and catalytic properties were synthesized. Diatomite was successfully inserted into LDH, and the DI-LDH sample exhibited strong catalytic activity for decreasing the CO and CO_2_ release during the thermal cracking of LDPE. Moreover, diatomite proved to be suitable for decreasing the degradation temperature and the release of CO_2_. The Cu/Al-LDH catalyst reduced the release of CO, while the DI-LDH catalyst combined the catalytic performance of diatomite and Cu/Al-LDH, which made it suitable for the pyrolysis of plastic materials. The synthesized DI-LDH catalyst may be a potential environmental catalyst for applications in the pyrolysis of LDPE waste by reducing the release of CO and CO_2_, which would greatly alleviate the white pollution caused by plastic products.

## Conflicts of interest

There are no conflicts to declare.

## Supplementary Material
